# Effectiveness of Photobiomodulation to Treat Motor and Non-Motor Symptoms of Parkinson’s Disease: A Randomised Clinical Trial with Extended Treatment

**DOI:** 10.3390/jcm14217463

**Published:** 2025-10-22

**Authors:** Anita E. Saltmarche, Orla Hares, Brian Bicknell, Ann Liebert, Margaret Naeser, Sujith Ramachandran, Jenna Sykes, Kaley Togeretz, Ashley Namini, Gillian Z. Heller, Geoffrey Herkes

**Affiliations:** 1 Saltmarche Health and Associates, Toronto, ON M4V 1T4, Canada; 2 Gaitway Neurophysio, Hamilton, ON L8P 2B6, Canada; orla@gaitwayneurophysio.com (O.H.); jennasykes00@gmail.com (J.S.); kaley@gaitwayneurophysio.com (K.T.); ashley.namini@gmail.com (A.N.); 3 Brain and Mind Centre, University of Sydney, Sydney 2050, Australia; brian.bicknell@sydney.edu.au; 4 College of Business, Recreation, and Education, Shepherd University, Shepherdstown, WV 25443, USA; ann.liebert@outlook.com; 5 Sydney Adventist Hospital, Sydney 2076, Australia; geoffery.herkes@sah.org.au; 6 Kolling Institute, University of Sydney, Sydney 2064, Australia; 7 Veterans Affairs Boston Healthcare System, Boston, MA 02130, USA; mnaeser@bu.edu; 8 Department of Neurology, Boston University School of Medicine, Boston, MA 02118, USA; 9 School of Pharmacy, University of Mississippi, Oxford, MS 38677, USA; sramacha@olemiss.edu; 10 NHMRC Clinical Trials Centre, University of Sydney, Sydney 2050, Australia; gillian.heller@sydney.edu.au; 11 College of Health and Medicine, Australian National University, Canberra 2601, Australia

**Keywords:** Parkinson’s disease, photobiomodulation, randomised controlled clinical trial, exercise, MDS-UPDRS, TUG, mobility

## Abstract

**Background/Objective:** Few treatment options improve symptoms and the quality of life of Parkinson’s disease (PD); more treatment choices are needed. This study examined the effectiveness of photobiomodulation therapy (PBMt) combined with exercise to improve PD symptoms and quality of life. **Methods:** Participants were randomised into Active (*n* = 32) or Sham (*n* = 31) PBMt groups. Stage 1 was an 8-week double-blind, randomised, placebo-controlled trial using either active or sham PBMt to the head, back of the neck and abdomen three times weekly at home, followed by a 4-week washout. Stage 2 was 8 weeks of active PBMt for all participants. In Stage 3, participants chose to continue active PBMt treatment (‘continuers’) or receive no PBMt treatment (‘non-continuers’) for up to 48 weeks. Participants continued vigorous exercise throughout the study. Participants were assessed on enrolment and after each stage. The primary outcome measure was timed up-and-go, with a range of secondary motor and non-motor outcomes, including UPDRS. **Results:** There was no significant difference between the Active and Sham Groups after Stages 1 or 2, apart from minimal increase in MoCA score/cognition (Sham Group) in Stage 1. After Stage 3, continuers showed a significant improvement in the primary outcome measure compared to non-continuers. Anxiety and the motor experiences of daily living (MDS-UPDRS Part II) were also significantly improved, while other outcomes approached significance, including MDS-UPDRS Total score (*p* = 0.062). **Conclusions:** As the largest study to date, results add increasing weight to previous clinical trials and highlight potential for at-home, scalable treatment as adjunctive therapy alongside medication and exercise.

## 1. Introduction

Parkinson’s disease (PD) is the second most common neurodegenerative disease, and its prevalence is increasing due to an ageing population, increased life expectancy and a confluence of environmental and personal factors [1]. The symptoms of PD invariably progress over time, as evidenced by increasing scores on the Movement Disorder Society—Universal Parkinson’s Disease Rating Scale (MDS-UPDRS) [2]. This is accompanied by a concomitant decrease in health-related quality of life (HRQoL) and an increasing social and economic burden. PD is also highly individual, characterised by patient-specific motor signs and non-motor symptoms, with each patient exhibiting their own constellation of symptoms, which results in substantial heterogeneity in individual symptom trajectory [3]. The pathology of PD involves the death of dopaminergic neurons and aggregation of α-synuclein. PD is routinely diagnosed based on the cardinal motor signs of resting tremor, bradykinesia, rigidity, and postural instability, but other motor signs, such as gait problems, freezing, dystonia, and akinesia, also contribute to the disease’s presentation. Non-motor symptoms, such as reduced sleep quality and REM sleep behaviour disorder, decreased cognition, and mood disorders (e.g., anxiety, depression), often have a greater influence on HRQoL than motor signs [4].

While medication can alleviate some symptoms, there is no pharmacological treatment that can halt or even slow disease progression or reverse motor signs [5] or non-motor symptoms [6]. Levodopa and dopamine agonists compensate for the loss of the dopamine neurotransmitter and can help reduce rigidity and bradykinesia but are less effective in addressing postural instability. Oral levodopa medication suffers from pronounced “off” periods, when dopamine is in short supply in the brain, and it also loses effectiveness as the disease progresses, requiring adjustments to medication levels. In addition, levodopa does not control non-motor symptoms; therefore, medication for these symptoms is initiated on a symptom-by-symptom basis, titrated according to individual need. Each medication is limited in its efficacy, and each carries its own list of side effects, some of which can be serious or even life-threatening [7]. As PD progresses, deep-brain stimulation may be considered. This will also not delay the progression of PD symptoms and carries its own retinue of side effects [8].

In addition to pharmaceutical and surgical interventions, several alternative treatment options are available, including device-assisted therapies [9]. Photobiomodulation therapy (PBMt) involves the use of red and near-infrared non-thermal light, delivered as either laser or LED, which can penetrate tissues to varying depths depending on the wavelength. PBMt acts by delivering photons to tissues, which are absorbed by cellular components (chromophores), thereby activating various cellular activities. One of the chromophores is cytochrome-C-oxidase (CCO) in the mitochondria. In PD, there is impaired mitochondrial energy metabolism. In compromised or hypoxic cells, mitochondrial ATP production is low. Photon absorption by CCO leads to a brief release of reactive oxygen species, an increased mitochondrial membrane potential, increased oxygen and increased ATP production, thereby boosting cellular energy and function [10]. Photon absorption also leads to the release of nitric oxide, stimulating vasodilation of blood and lymphatic vessels [11]. Other responses include the activation of signalling pathways, such as transcription factors and changes in gene expression, including Glial-Derived Neurotrophic Factor (GDNF) and brain-derived neurotrophic factor expression (BDNF) [12]. These events promote cell survival, cellular growth, repair, and inhibit inflammatory processes. Extensive research has demonstrated the anti-inflammatory effects of PBMt across a variety of acute and chronic diseases and disorders [13]. Chronic inflammation and the body’s immune system response play an important role in PD [14,15,16], with both peripheral inflammation and neuroinflammation contributing to the onset and progression of the neurodegenerative processes seen in PD [17,18,19]. This suggests that the interaction between the gut and the brain is not only important in maintaining brain health, but there may also be a link between gut dysbiosis and neurodegenerative and neurodevelopmental diseases [19].

Over 40 years of clinical and research evidence, as well as a recent Delphi consensus, have established the safety and efficacy of PBMt for various musculoskeletal, wound, and pain conditions. There is substantial pre-clinical evidence that PBMt can reduce the motor signs of PD [20,21], and that it is both neuroprotective and neurodegenerative in rodent and non-human primate models of PD [22,23]. This effect can be achieved whether the PBMt is delivered to the head or to body areas remote from the head, primarily the abdomen [24]. In clinical trials [25], PBMt has shown a positive impact on motor signs and non-motor symptoms of Parkinson’s disease in proof-of-concept trials [26,27], case studies [28,29] and in a previous randomised placebo-controlled clinical trial (RCT) [30,31]. This impact has been observed with PBMt directed to the head alone [30,31], directed to the abdomen and neck without directing light to the scalp/brain [26] or using a combination of targets [27]. The choice of PBMt targets was based on numerous pre-clinical studies on mice, rats and monkeys [20,24]. It has also been demonstrated that the improvements in PD symptoms can be sustained for up to five years with continued PBMt [29,32].

Exercise, particularly exercise targeted for PD, can also have a positive impact on symptoms [33,34]. High-intensity exercise regimens [35] such as Rock Steady Boxing [36], and the PDWarrior exercise program [37] appear to influence Parkinson’s motor signs and HRQoL, although the longer-term effects are unknown.

The above PBMt clinical trials and real-life clinical experience have demonstrated that a multi-modal approach, such as PBMt, combined with pharmacological intervention and targeted exercise, may provide the best opportunity to positively impact the motor signs and non-motor symptoms associated with PD. Therefore, this study incorporated PBMt into an already established program that included targeted exercise programs for Parkinson’s disease symptoms.

### Objective

The objective of this study was to examine whether there were significant differences in motor, non-motor and HRQoL symptoms in participants who received 8 weeks of active PBMt combined with exercise compared to those who received sham PBMt plus exercise. A second objective was to determine the impact of PBMt with extended treatment.

## 2. Materials and Methods

### 2.1. Regulatory Approval

The study was approved by the Scientific Review Committee at Advarra (# Pro00060895) Columbia, MD, USA, and obtained a Health Canada Investigational Testing Authorisation (ITA) approval, application number 347697, Ottawa, ON, Canada. The study was registered with https://clinicaltrials.gov/study/NCT06036433?cond=NCT06036433&rank=1 (The registration date: 30 August 2022, NCT code NCT06036433). Montreal Cognitive Assessment (MoCA), version 8.1, Greenfield Park, QC, Canada. Movement Disorder Society—Unified Parkinson’s Disease Rating Scale (MDS-UPDRS), 2019, Milwaukee, WI, USA.

### 2.2. Recruitment

Participants were recruited from the Parkinson’s Wellness Innovation Centre located in Hamilton, Ontario, Canada, where targeted exercise classes took place as well as from local PD support and exercise groups. Subjects with neurologist-diagnosed Hoehn and Yahr Stages 2–3 (moderate) idiopathic PD, with or without anti-Parkinson’s disease medications, were screened using the inclusion/exclusion criteria in Supplementary Table S1. Participants gave written informed consent when enrolled.

### 2.3. Study Design

The number of participants was calculated using the Timed Up & Go test (TUG) to evaluate a person’s mobility, requiring both static and dynamic balance. Based on previous data, we estimated that the mean TUG time after 8 weeks of treatment will be 7.7 seconds with a standard deviation of 2.64. Assuming an effect size of 0.88 [26,27] a power of 80% and an alpha of 0.05 (two-sided), we will require 25 patients per group, or a total of 50 subjects to be enrolled in the study. To ensure that enough participants complete the study, 60 participants were recruited to account for potential dropouts, and any initial dropouts were replaced.

**Figure 1:**
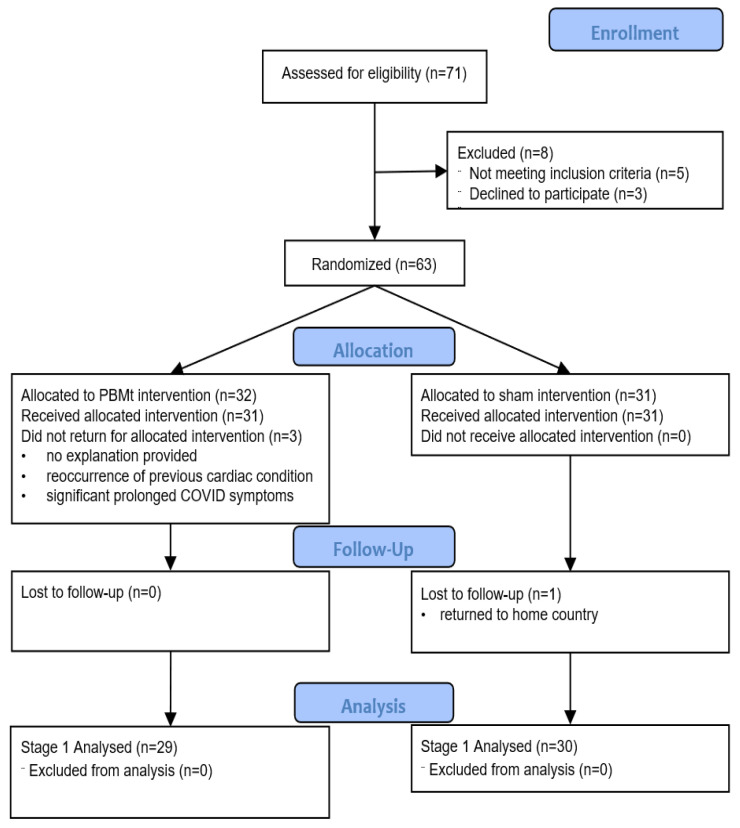
Consort Flowchart for Stage 1 (Randomised, Controlled Trial Stage).

**Figure 2:**
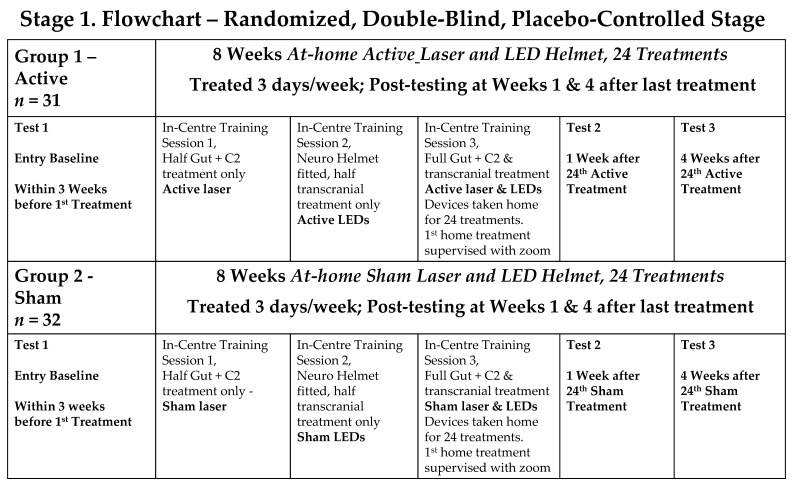

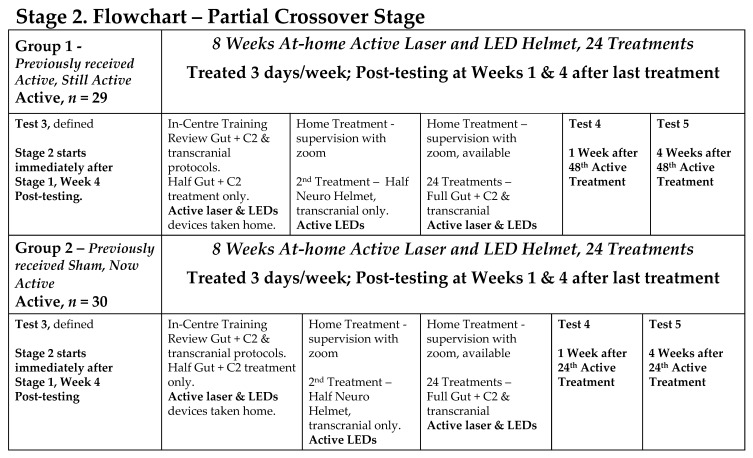

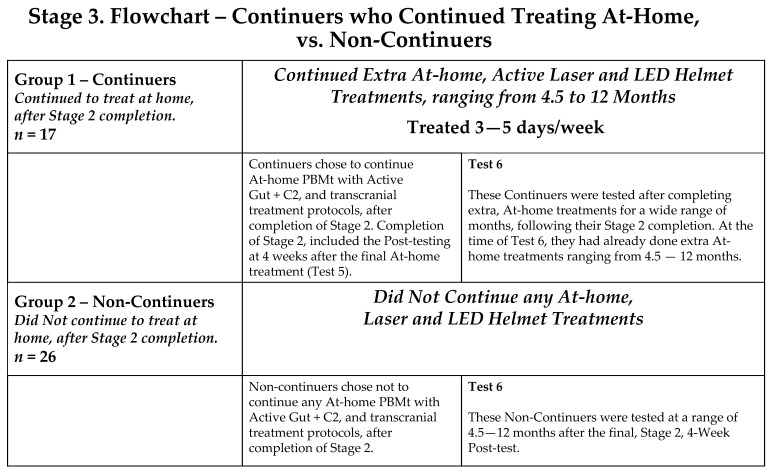
Study Flowcharts.

The study adhered to the CONSORT guidelines (Figure 1) and consisted of three stages, which are summarised in Figure 2.

- Stage 1: a double-blind, randomised, placebo-controlled trial (RCT), with participants randomly divided into an Active PBMt group and a Sham PBMt group. Participants in the Sham Group were informed that the LED helmet device only produced near-infrared light, invisible to the naked eye. The only researchers who knew group allocation were a statistician (SR) and researchers who trained the participants in at-home use of PBMt (AS, JS, KT). Outcome assessments were conducted by the neurophysiotherapist (OH), who was blinded to the treatment groups.
- Stage 2: a partial cross-over where all participants received active PBMt.
- Stage 3: participants had the choice of continuing at-home PBMt (“continuers”) or not continuing PBMt (“non-continuers”).

There was a variable length of time between the end of Stage 2 and the beginning of Stage 3, and for the duration of continued Active PBMt within Stage 3. All participants continued to exercise throughout the study.

### 2.4. Photobiomodulation Therapy Intervention

**Figure 3:**
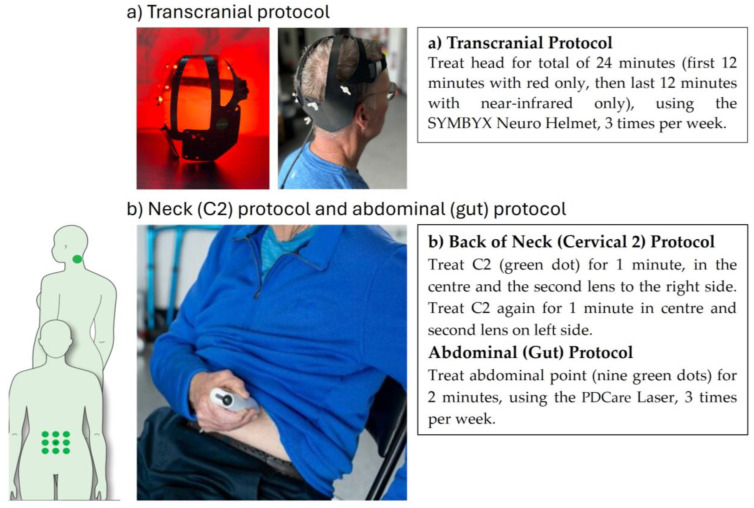
(**a**,**b**) Photobiomodulation Intervention Protocols.

PBMt consisted of a two-part intervention. Transcranial PBMt used an LED helmet device (the SYMBYX Neuro—Figure 3a), with 20 separate LED clusters, each containing one near-infrared (810 nm) and one red (635 nm) LED. Transcranial treatment targeted the brain to decrease inflammation, increase microcirculation and glymphatic drainage. The SYMBYX Abdomen-Neck Protocol used the PDCare laser, a near-infrared, class 1 laser device (‘PDCare Laser’—Figure 3b) with two superpulsed, 904 nm diodes. Light was delivered to nine points on the abdomen and the back of the neck (C2/C3 level). The application of PBMt to the upper cervical spine was to affect C2 and C3 dorsal root ganglion connectome to trigeminal nucleus and the putative Endorestiform Nucleus [38]. This was further verified by two other small clinical trials using remote PBMt for treating Parkinson’s [26,27]. Abdominal PBMt treatment has been shown to result in neuroprotective effects in pre-clinical PD models and may trigger systemic effects, supporting the immune system. Sham devices were identical but emitted no light. Device parameters and PBMt dosages are given in Supplementary Table S2. The treatments took 24 min and were conducted three times a week, with at least a 48-h interval between treatments.

In Stages 1 and 2, the first three PBMt sessions were in-clinic. The first treatment was a “test dose” (half dose) of the SYMBYX Abdomen-Neck Protocol using the PDCare 904 laser intervention on the back of the neck and on the abdomen. The second was a test dose of transcranial PBMt. The third was a self-administered complete treatment. In addition to assuring the safety of the PBMt, the sessions served as treatments for the first two stages. In Stage 3, at-home treatment was less constrained. Some participants increased the frequency of transcranial PBMt, and the total number of PBMt treatments varied among participants. Participants were instructed not to make any changes to their medications, exercise levels, or diets during the study.

### 2.5. Participant Contact, Support and Safety

Participants were contacted weekly by phone or email to document adherence to the at-home PBMt protocols, to address any issues or answer questions as needed, and record exercise, sleep patterns, bowel movements, and any changes to medications and/or symptoms. Any suspected adverse event (SAE), side effect of PBMt or reaction to the treatment was documented and passed on to the SAE advisory panel (blinded to the groups), who then decided on appropriate courses of action.

### 2.6. Outcome Measurement Assessments

Participants were assessed (Figure 2) at baseline (no more than 3 weeks prior to the first treatment), at 1 and 4 weeks after Stages 1 and 2, and after Stage 3. The primary outcome measure was Timed Up-and-Go (TUG). Secondary outcome measures included assessment of gait and mobility, dynamic balance, fine motor skills, cognition, mental health, sleep quality, quality of life and the MDS-UPDRS assessment. Outcome measures are listed in Table 1. All outcome measures were assessed by one Clinical Investigator (OH) with extensive PD experience, who was blinded to treatment groups and was not involved in data analysis. Further outcome measures, such as changes in voice, facial expression, and writing ability (micrographia), as well as qualitative HRQoL data, are the subject of a future manuscript.

### 2.7. Statistical Analysis

Treatment efficacy was evaluated at entry baseline, at one week and four weeks after the end of Stages 1 and 2 and once in Stage 3. In Stages 1 and 2, ANCOVA was used to compare the scores for the outcome measures between the Active and Sham Groups. For the Stage 1 analysis, the post-testing scores were compared to the entry baseline. For the Stage 2 analysis, the post-testing scores at 1 week after the final treatment in Stage 2 were compared to an ‘adjusted’ baseline mean consisting of the entry baseline and the 1-week post-testing score from Stage 1 for each outcome measure. Week 4 measurements were not used in the analysis.

In Stage 3, ANCOVA was used to compare the scores for each group (continuers and non-continuers) for each outcome measure obtained only once in Stage 3. The entry baseline scores and the measurements at the start of Stage 3 (e.g., the scores at 1 Week Post Stage 2) were used in the ANCOVA. Then, the ANCOVA compared the Stage 3 measurements between the continuers and non-continuers.

For outcome variables that were not normally distributed (TUG, TUG-dual Task, TUG-cognitive), a log transformation was applied. No imputation was used. A significance level of 5% was used with no adjustment for multiple comparisons. Suspected adverse events and symptoms were also evaluated.

## 3. Results

The first participant was enrolled in the study on 2 January 2023, and the final data were collected on 1 February 2025. Sixty-three male (*n* = 43) and female (*n* = 20) participants aged between 58 and 79 years old met the inclusion/exclusion criteria and were randomised into the Active or Sham Group. The groups were balanced in demographic features and symptoms (Supplementary Tables S3 and S4), except that there was a higher percentage of females in the Active Group (44%) compared to the Sham Group (19%). Of the 63 participants who entered the study, there were four withdrawals during Stage 1, eight during Stage 2, and a further eight did not continue into Stage 3 (Supplementary Table S5). Of the 43 subjects in Stage 3, 17 were continuers and 26 were non-continuers.

Three of the withdrawals might have potentially been related to the PBMt as determined by the SAE advisory committee: one with self-reported atrial fibrillation and headaches, one with a recurrence of vivid dreams and one who reported hair loss. The SAE advisory panel did not require any participant to be withdrawn from the study. There were no major side effects during the study. Additionally, there were no withdrawals or side effects after extended PBMtt over 12–48 weeks of treatment during Stage 3 of the study.

**Figure 4:**
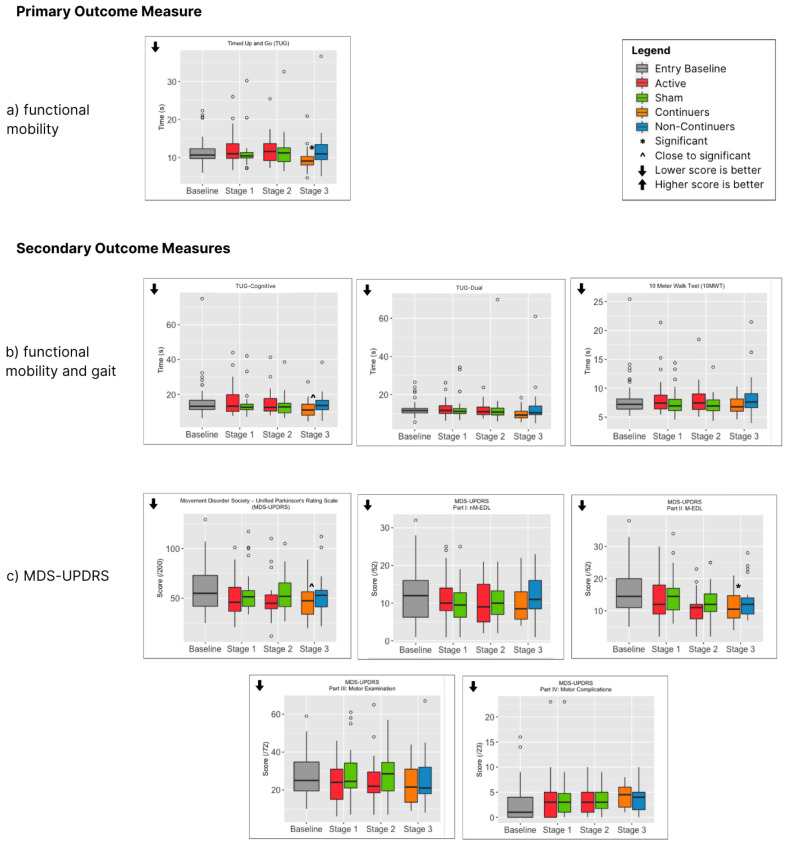

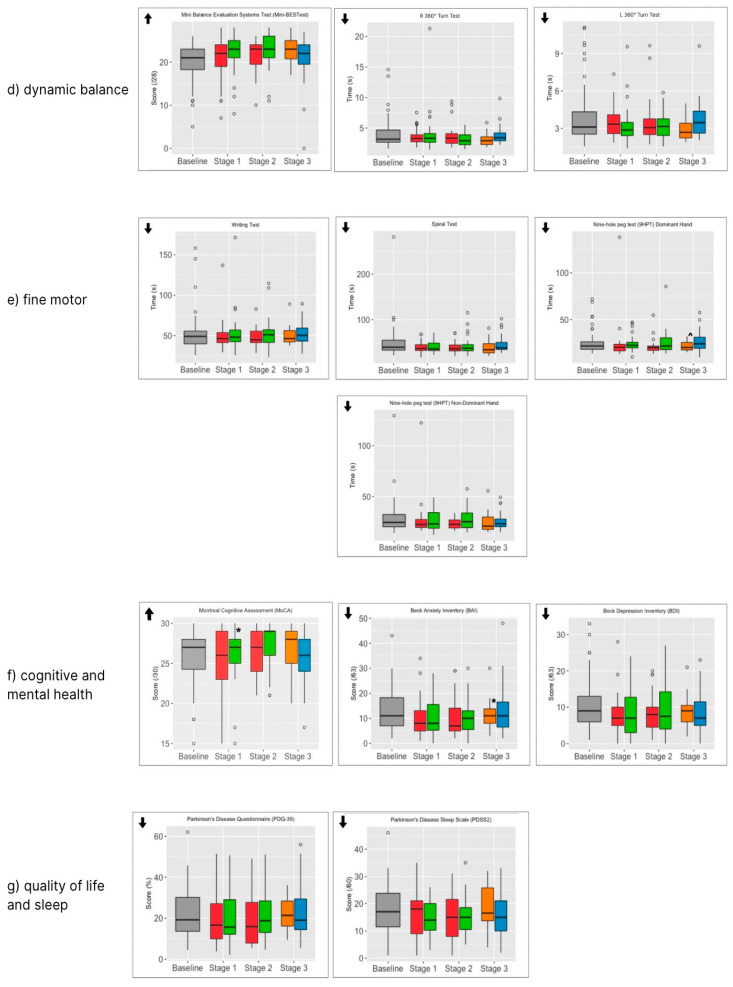
Primary and Secondary Outcome Measure Box Plots (showing medians and IQR).

No outcome measure showed a significant difference between active and sham in Stages 1 or 2 (Table 2, Figure 4) apart from the Montreal Cognitive Assessment (MoCA), which showed a greater improvement in the Sham Group Stage 1. Compared to baseline, the primary outcome (TUG) showed no improvement during Stage 1 or Stage 2 (Figure 4a). However, several secondary outcomes did improve with active PBMt during Stages 1 and 2, the most noteworthy being the MDS-UPDRS total score (Figure 4c), with an average improvement (decrease) of 7.9 points after Stage 1 (active PBMt) and 10.5 points after Stage 2 (active PBMt then active PBMt) (Table 2). Other secondary measures to improve included the M-EDL and motor examination components of the MDS-UPDRS, sentence writing, time to complete the 9 Hole Peg Test, PDQ39 and anxiety (Table 2, Figure 4c,e–g).

**Figure 5:**
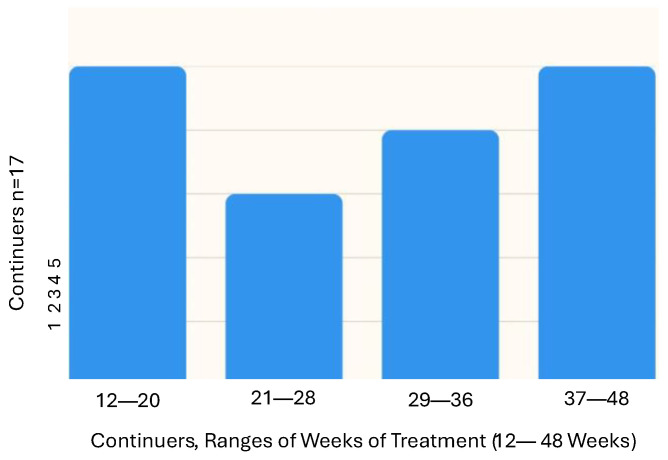
Number of Weeks of Treatment for Continuers at Stage 3 Testing.

For those participants who were tested after Stage 3, the total number of active PBMt treatments varied according to whether they were part of the Sham or Active PBMt Group (Stage 1), whether they did or did not continue with PBMt (Stage 3) as well as the number of weeks of PBMt for continuers in Stage 3 (Figure 5). Stage 3 lasted from 4.5 to 11 months, with 81% of participants between 24 and 36 weeks, with an average of 28.7 weeks of treatment. After Stage 3, continuers showed a significant difference in the primary outcome measure (TUG) compared to non-continuers (Figure 4a and Table 2). There was also a significant difference in two secondary outcomes, MDS-UPDRS-II (mEDL) and the Beck Anxiety Inventory (Figure 4c,f). Several other secondary outcomes approached significance (*p* < 0.1), most notably the MDS-UPDRS total score (*p* = 0.062), but also TUG-Cognitive (*p* = 0.058) and the 9 Hole Peg—dominant hand (0.087). It is also noteworthy that almost all outcome measures showed more improvement for continuers compared to non-continuers (Table 2, Figure 4), the exceptions being the Beck Depression Inventory, MDS-UPDRS-IV (motor complications) and the Parkinson’s disease Sleep Scale (PDSS2).

## 4. Discussion

The use of PBMt was demonstrated to be safe, with only transient minor reactions reported. Of the 20 participants who withdrew from the study, only three might have been due to the effects of PBMt. One had symptoms of headache and nausea that were associated with a cardiac issue. This participant had a history of cardiac arrhythmia and had requested entry into the study since her symptoms had improved. This would have typically excluded her from the study. This person was not included in the data analysis. It is questionable as to whether the symptoms of the other two (hair loss and the recurrence of vivid dreams) were due to the PBMt. The other minor transient reactions to PBMt reported here are consistent with other studies of transcranial PBMt [53,54].

In the randomised placebo-controlled stage of the study (Stage 1), the MoCA showed a significant difference between those who received active treatment and those who received sham treatment in Stage 1, but this was not repeated in Stage 2 or Stage 3. This difference was not considered to be clinically relevant. No other outcome measure showed a significant difference between Active and Sham Groups in Stages 1 and 2. Several outcome measures, however, showed a non-significant improvement over the 8 weeks of Stage 1 compared to the entry baseline, both for Active and Sham Groups. These included the MDS-UPDRS total score and some of its parts (M-EDL and nM-EDL), as well as balance (Mini-BESTest). There was, however, no improvement in the primary outcome measure (TUG). The improvements made during Stage 1 generally continued through Stage 2, where all participants were using Active PBMt devices.

The reasons for this lack of difference between PBMt plus exercise and exercise alone in Stages 1 and 2 could be the well-documented placebo effect that occurs when patients become part of a Parkinson’s clinical study [55,56,57]. In addition, the exercise that all participants undertook, the positive social environment during the exercise classes during the study, and the relatively short 8-week intervention period could have contributed to the lack of difference. Exercise is known to help with the symptoms of Parkinson’s disease and cognition, at least in the short term. Reviews of clinical studies that included exercise have shown a positive effect for motor signs of Parkinson’s disease [33,58] and especially for exercise regimens targeted for Parkinson’s patients, such as Rock Steady Boxing [36], PDWarrior [37], and Park-in-Shape [59]. These exercise regimens have been shown to result in improvements in MDS-UPDRS-III (motor examination) scores and quality of life (PDQ-39). We have also previously seen improvements in Parkinson’s symptoms that have lasted several years with continued PBMt and exercise [29]. Many of the participants in the current study initiated a more regular exercise regimen upon enrolling in a newly established facility that fostered collegiality in a friendly, social setting, which may also have had an impact on their Parkinson’s symptoms [60].

The significant difference in Stage 3 of the primary outcome measure (TUG) in continuers was a demonstration of the positive effect of the additional weeks of PBMt. This was supported by the significant difference in m-EDL, as well as the difference in MDS-UPDRS total score and other differences that approached significance (such as TUG cognitive). This highlights the fact that long periods of treatment may be necessary for the positive effect of treatment of PD with PBMt to become apparent. As a further indication that long-term PBMt was effective, the majority of secondary outcome measures showed improvement in continuers compared to non-continuers, and the mean of most outcome measures was improved after Stage 3 compared to the entry baseline when PBMt was continued. This included TUG, which had not improved above baseline during Stage 1 or 2. Collectively, the results indicate that PBMt has a safe and positive effect on both motor signs and non-motor Parkinson’s symptoms, provided treatment is continued for an extended period. This supports that PBMt should be incorporated into the management of Parkinson’s disease as an additional treatment option.

Mobility and gait, as reflected by the three measures of TUG, the walk test and the balance tests, all show improvements over time and improvements in continuers compared to non-continuers. This improvement is meaningful, given that Parkinson’s disease is often primarily diagnosed with motor signs, as well as the impact that mobility has on PwP and caregivers. In addition to motor signs, the PDQ39 measure of HRQoL improved, and the measure of anxiety showed a significant reduction in continuers compared to non-continuers. Arguably, the improvement in non-motor symptoms is more important than that seen in motor signs, since these often have a greater influence on HRQoL. The importance of non-motor symptoms is often underestimated or downplayed in the medical management of Parkinson’s disease, but these changes can be more debilitating than motor signs [61] and are more problematic to treat pharmacologically. HRQoL is often considered by people with Parkinson’s and their caregivers, family and others as being more important than the patients’ mobility [62]. Qualitative measures of quality of life are explored in a further publication.

The reduced effect of PBMt in non-continuers may be due to the total number of treatments that they received, with PBMt treatments needing to pass beyond a threshold for significant improvement. Non-continuers received either 9 or 18 weeks of treatment, depending on whether they were in the Sham or Active Group, while continuers received an average of 28.7 weeks of treatment. The difference between the groups is most likely, however, related to the combination of the number of weeks of treatment and the time between their last treatment in Stage 2 and their Stage 3 assessment. As a slowly progressing neurodegenerative disease, it is perhaps unsurprising that it could take up to one year before the difference between PBMt and non-treatment becomes apparent. For example, any placebo effect might last for some months [57], and it has been reported that exercise specifically targeted to Parkinson’s disease can have an impact on symptoms for some months [35]. Previously reported research demonstrated that the positive effect of PBMt on PD symptoms might take months to become apparent [30]. Indeed, individual participants in our original proof-of-concept PBMt study [27] showed that while some participants responded relatively quickly to the treatment (within weeks), others continued to improve gradually in outcome measures such as static balance and TUG over a year or more. This improvement, once achieved, was shown to last for up to 5 years with continued treatment [32]. Other studies using PBMt for intractable chronic diseases have also shown that positive results may take an extended time to become apparent and statistically significant. For example, in a fibromyalgia study, there was a sustained reduction in pain at 6 months but not 3 months following 12 weeks of whole body PBMt [63]. Fibromyalgia-type pain is common in Parkinson’s disease [64].

Transcranial PBMt has been shown to have potential for the treatment of several neurological conditions, such as traumatic brain injury, Alzheimer’s disease, stroke and neuropsychiatric conditions [65]. In the study reported here, the placement of LEDs in the transcranial helmet is concentrated towards the back of the head and upper neck region, with the putative Endorestiform Nucleus as a target [38]. This area is thought to integrate sensory and motor information and may have relevance to PD. A study using this helmet alone has also shown promising results for PD motor signs [28,29], despite red and near-infrared light being incapable of penetrating directly to the depth in the brain required to reach the areas affected by the lack of dopamine. The targeting of PBMt to the abdomen and neck has also been shown to alter PD symptoms, even in the absence of transcranial PBMt, in both animal models [22] and clinically [25]. Remote PBMt that targets the abdomen and leg has demonstrated neuroprotective effects and comparable results to transcranial treatment in both mouse and non-human primate models of Parkinson’s [22]. While the mechanism of action of remote PBMt is not fully understood, there are several hypotheses. Indirect PBMt may trigger systemic or abscopal effects, via chemical messengers or inflammatory signals, which influence remote tissues [66]. Another proposed mechanism relates to the gut–brain axis, wherein the abdomen is treated to elicit effects in the brain via the microbiome or otherwise from the enteric nervous system and the vagus nerve [24,25,67]. We have previously shown changes in the microbiome with either transcranial plus abdominal PBMt [68] or with abdominal PBMt alone [26], which may be maintained for up to 5 years [69]. The combined approach of targeting the abdomen as well as the head may assist in influencing deeper areas of the brain that have been affected by a lack of dopamine.

We might hypothesise that the prolonged time to see a significant difference between the continuing and non-continuing groups is due to the length of time that it takes for neuroplasticity to occur in order to compensate for the deterioration in the dopaminergic neurons and lack of dopamine, perhaps analogous to the reorganisation of neural networks after a traumatic brain injury [70]. Neuroplasticity is known to occur naturally in PD [71], with the initial response to dopaminergic neuron loss being an increased efficiency of surviving neurons and the consequent masking of symptoms for some years. PBMt has been shown to be potentially neuro-regenerative in animal models [72] and the potential role of PBMt in neuro-regeneration in humans deserves further exploration.

Results for the effect of PBMt on Parkinson’s symptoms are highly individual, as would be expected for a disease with such heterogeneous symptomology. Multiple factors can influence the presentation of symptoms, including genetic predisposition, stage of the disease, and the postulated body-first and brain-first variants of the disease [73]. It should also be noted that at baseline, the majority of participants in this study were categorised as mild to moderate for most symptoms (Hoehn and Yahr stage 2), thus leaving less room for improvement than in more advanced stages of PD, where response to PBMt might be different.

The study has several limitations. The number of participants was relatively small, the potential impact of medication was not reviewed, and the 8-week treatment period for the RCT stage was relatively short, considering the nature of the neurodegenerative disease and its potential for a marked placebo effect, and no imaging. Multiple statistical tests raise the level of significance into question, but this is offset by the ability to capture potential changes across various Parkinson’s symptoms. Participants engaged in a range of exercise types. A key limitation is the variability in the total number of PBMt treatments completed by each participant, which reflects real-life clinical practice and research. Future research may want to consider a larger sample size, a longer treatment period, a higher total number of treatments, and incorporate one specific type of exercise.

## 5. Conclusions

In conclusion, this is the largest study to date that has assessed the efficacy of PBMt as a treatment for Parkinson’s symptoms and increases the weight of evidence of the efficacy of this device-assisted therapy. There was a significant difference in the primary outcome measure (TUG) when PBMt was continued for an extended time. Notably, there was also a significant improvement in anxiety for those who continued PBMt. Longer term, larger studies are warranted from the results presented here, with the intention to slow the progression of Parkinson’s symptoms.

As a real-world, pragmatic clinical study, the importance of a multidisciplinary approach to treating the complexity of Parkinson’s disease symptoms is emphasised. PBMt is a cost-effective and home-based adjunct therapy that can be used alongside pharmacological interventions, exercise, social contact and personal interactions to improve PD symptoms. Importantly, PBMt has the potential to be a scalable therapy to meet the increasing demand for Parkinson’s services without compromising quality or efficiency. As a safe, self-administered, home-based treatment, PBMt, combined with clinical support, can be accessible to those living in regions with minimal specialised services and in remote rural settings requiring minimal healthcare resources.

The ultimate aim of the series of studies on PBMt for PD is to establish guidelines for its use as part of standard care in PD, in the same way that PBMt now has medical guidelines for its use in mitigating side effects of radiation and chemotherapy in cancer treatment (https://mascc.org/resources/mascc-guidelines/ (2019–2020) (accessed on 10 September 2019) Healthcare professionals working with this population should be aware of and knowledgeable about PBM’s safety history and potential effectiveness in treating Parkinson’s disease symptoms within a multidisciplinary approach, enabling them to guide patients’ treatment.

## Figures and Tables

**Table 1:** Outcome Measures.

Outcome Measure	Test	Description	Interpretation
Primary Outcome Measure			
Functional mobility	Timed Up and Go (TUG)	Assessors measured the time taken for a participant to stand from a chair, walk 3 m, turn around a marker, return and sit down [39]	A quicker time is better. ≥14 s is indicative of falls risk [39]
Secondary Outcome Measures			
Functional mobility	TUG-Dual	As for TUG, except that the participant was carrying a cup of water [39]	A quicker time is better. ≥14 s is indicative of falls risk [39]
	TUG-Cognitive	As for TUG, except that the participant was asked to count backwards from 40 by twos [39]	A quicker time is better. ≥4 s is indicative of falls risk [39]
Gait	10 m walk Test (10MWT)	Participants walked a straight 10 m track. After walking 2 m, assessors measured the time taken to walk a further 6 m and the number of strides taken [40]	A faster speed is better. <1.1 m/s is indicative of falls risk Fewer strides is better
Neurological assessment	MDS-UPDRS	Evaluation of various aspects of Parkinson’s disease by a trained MDS-UPDRS assessor (OH), including non-motor and motor experiences of daily living, motor complications, and burden of disease [41]. MDS-UPDRS Parts I, II, III and IV were also separately reported	A lower score is better. Part I 10 and below is mild Part II 12 and below is mild Part III 32 and lower is mild Part IV 32 and below is mild
Dynamic balance	Mini-BESTest	A balance test consisting of 14 items, including tasks divided into four subcomponents: anticipatory postural adjustments, postural responses, sensory orientation, and dynamic gait [42]	A higher score is better.
	360° Turn test	Participants turn 360 degrees, and assessors measure the time taken and number of steps to complete the turn [43]	A faster speed is better Fewer steps is better
Fine motor skills	Nine-hole peg test (9HPT)	Assessors recorded the time taken for participants to place 9 pegs in holes and then return the pegs to the reservoir. Both hands were tested [44]	A faster time is better
	Writing Test	Participants wrote the same sentence at each assessment. The time taken to complete the sentence was recorded	A faster time is better
	Spiral Test	Participants drew a spiral within a template on a sheet of paper, and assessors recorded the time taken to complete the task	A faster time is better
Cognition	Montreal Cognitive Assessment (MoCA)	Participants completed the MoCA test version 8.1 (www.mocatest.org (accessed on 15 October 2022)), which was scored by a trained assessor [45]	A higher score is better (max 30). ≤25 may indicate mild cognitive decline
Mental health	Beck Depression Inventory (BDI-II)	Participants completed a self-report questionnaire of 21 items, rating characteristic attitudes and symptoms of depression [46]	A lower score is better ≥13 is indicative of minimal depression 14–19 is indicative of mild depression [47]
	Beck Anxiety Inventory (BAI)	Participants completed a self-report questionnaire of 21 items, rating common somatic and cognitive symptoms of anxiety [48]	A lower score is better (max 63) ≤7 is indicative of no anxiety 8–15 is indicative of mild anxiety [49]
Quality of Life and activities of daily living	Parkinson’s disease questionnaire (PDQ39)	Participants completed a 39-item self-report questionnaire that assesses how often they experience difficulties across eight dimensions of daily living, including relationships, social situations, and communication. It also assesses the impact of Parkinson’s on specific dimensions of functioning and wellbeing [50]	A lower score indicates a better QoL
Sleep quality	Parkinson’s disease sleep scale (PDSS2)	Participants completed a self-report questionnaire of 10 questions that assesses the level of sleep disruption being experienced [51]	A lower score is better. A score ≥ 18 may indicate clinically relevant PD-specific sleep disturbances [52]

**Table 2:** Comparison of outcome measures [mean, (sd)] at baseline and after stages 1, 2, and 3, showing ANCOVA *p*-values where significant or approaching significance.

	Entry Baseline	Stage 1		Stage 2		Stage 3	
Outcome Measures	(*n* = 63)	Active (*n* = 29)	Sham (*n* = 30)	Active Then Active (*n* = 24)	Sham Then Active (*n* = 27)	Continuers (*n* = 17)	Non- Continuers (*n* = 26)
Primary Outcome Measure							
TUG time	11.4 (3.2)	12.3 (4.2)	11.0 (4.0)	12.0 (4.0)	11.6 (4.8)	9.8 (3.8)	12.0 (5.6)
						*p* = 0.016 *	
Secondary Outcome Measures							
TUG-dual time	12.1 (3.2)	12.7 (4.3)	12.0 (5.1)	12.3 (3.9)	13.1 (11.6)	10.2 (3.4)	13.5 (10.3)
TUG-cognitive time	15.4 (9.2)	16.6 (8.9)	13.6 (6.1)	15.6 (7.7)	13.6 (6.6)	12.3 (5.7)	14.6 (6.5)
						*p* = 0.058 ^	
MDS UPDRS total score	59.8 (22.1)	51.9 (21.5)	55.9 (21.0)	49.3 (20.6)	54.4 (19.8)	47.8 (19.4)	53.1 (19.6)
						*p* = 0.062 ^	
MDS-UPDRS-I	12.1 (7.3)	11.5 (6.0)	10.3 (5.3)	10.1 (5.9)	10.2 (4.8)	10.0 (5.4)	11.7 (5.4)
MDS-UPDRS-II	16.4 (6.9)	13.8 (7.7)	14.5 (6.4)	10.7 (4.6)	12.7 (5.7)	11.2 (5.0)	13.0 (5.3)
						*p* = 0.048 *	
MDS-UPDRS-III	28.1 (11.2)	24.4 (9.3)	27.7 (12.8)	24.1 (12.7)	24.4 (10.2)	22.9 (11.3)	24.9 (12.3)
MDS-UPDRS-IV	2.7 (3.5)	3.9 (4.8)	3.7 (4.4)	3.3 (2.8)	3.6 (2.6)	4.3 (2.4)	3.4 (2.3)
10MWT time	7.9 (3.0)	8.4 (3.4)	7.4 (2.0)	8.1 (2.8)	7.0 (1.8)	7.1 (0.2)	8.4 (3.5)
Right 360° turn test time	4.1 (2.4)	3.7 (1.4)	3.5 (1.5)	3.9 (2.1)	3.1 (1.1)	3.2 (1.1)	3.8 (1.6)
Left 360° turn test time	3.9 (2.2)	3.6 (1.3)	3.1 (1.1)	3.7 (1.9)	3.2 (1.1)	3.0 (0.9)	3.7 (1.6)
Sentence write time	51.3 (18.7)	50.7 (19.6)	49.8 (13.3)	47.9 (12.9)	52.9 (20.2)	50.7 (12.7)	51.4 (14.8)
Spiral draw time	49.4 (35.5)	39.4 (12.6)	41.0 (13.5)	39.6 (13.4)	44.2 (20.9)	31.0 (17.5)	46.7 (19.3)
9HPT dominant hand	24.8 (11.5)	24.6 (22.4)	24.5 (8.6)	21.7 (8.7)	26.0 (14.0)	21.4 (5.0)	26.9 (10.7)
						*p* = 0.087 ^	
9HPT non-dominant hand	28.9 (16.1)	27.2 (19.2)	27.0 (10.0)	23.2 (5.1)	27.6 (11.0)	25.5 (10.9)	25.9 (8.9)
MoCA	26.0 (3.4)	25.6 (3.7)	26.4 (2.8)	26.4 (2.9)	27.5 (2.7)	27.1 (2.8)	25.6 (3.2)
			*p* = 0.015 *				
Beck depression	10.8 (7.0)	8.0 (5.5)	8.7 (6.4)	8.4 (5.8)	9.3 (7.0)	9.3 (4.6)	8.7 (5.8)
Beck anxiety	13.2 (8.4)	10.5 (7.8)	9.9 (6.7)	10.6 (8.1)	11.2 (7.8)	11.8 (6.5)	14.0 (10.4)
						*p* = 0.050 *	
PDQ39	22.2 (12.2)	20.0 (13.5)	21.4 (13.4)	18.3 (11.9)	21.6 (12.3)	22.0 (8.5)	24.1 (13.8)
PDSS2	18.1 (8.9)	16.1 (8.1)	14.6 (6.5)	14.7 (8.3)	15.2 (7.2)	18.8 (8.7)	15.7 (7.5)

Note: The means (sd) are based on Week 1 post-testing. Abbreviations: TUG, timed up-and-go; 10MWT, 10 metre walk test; MDS-UPDRS, Movement Disorder Society—Unified Parkinson’s disease rating score; M-EDL, motor experiences of daily living; nM-EDL, non-motor experiences of daily living; PDQ, Parkinson’s disease questionnaire; PDSS, Parkinson’s disease sleep score; MoCA, Montreal cognitive assessment; 9HPT, 9-hole peg test. * = significant difference (*p* ≤ 0.05); ^ = approaching significance (*p* < 0.10).

## Data Availability

Data will be available upon request and approval from the IRB.

## Supplementary Materials

The following supporting information can be downloaded at: https://www.mdpi.com/article/10.3390/jcm14217463/s1, Table S1: Inclusion/Exclusion Criteria; Table S2: Treatment Parameters; Table S3: Demographic and Characteristics of Study Participants at Baseline; Table S4: Participant Symptom Characteristics at Baseline; Table S5: Study Withdrawals.

## Abbreviations

The following abbreviations are used in this manuscript:

PBMt	Photobiomodulation treatment
PwP	People Living with Parkinson’s
HRQoL	Health Related Quality of Life
PD	Parkinson’s Disease
