# Supplementary material for: Effectiveness of Photobiomodulation to Treat Motor and Non-Motor Symptoms of Parkinson’s Disease: A Randomised Clinical Trial with Extended Treatment
Source: J Clin Med. 2025 Oct 22;14(21):7463. doi: 10.3390/jcm14217463 (PMC12609809; doi:10.3390/jcm14217463)
Supplement: Supplementary file 1 [file jcm-14-07463-s001.zip › jcm-3877390 Supplementary Materials.pdf]

Supplementary Materials

Table S1: Inclusion/Exclusion Criteria

- Females and males aged 55-79 years;
- Neurologist-diagnosed Hoehn and Yahr Stages 2-3 (moderate) idiopathic PD;
- With or without anti-Parkinson's Disease medications; with stable usage for past six months;
- Ability/ willingness to attend the PD Wellness & Innovation Centre in Hamilton, Ontario, Canada, for PBMt teaching/ supervision sessions and administration of outcome measures (total of 9 in-person sessions, including screening and baseline outcome measurement testing, four teaching sessions across Stages 1 and 2, and five outcome measurement testing appointments, two at Stage 1 weeks 1 & 4 post treatment, one at Stage 2, Week 4 post treatment session and Stage 3 outcome measurement testing;
- Internet access for video training sessions or supervision of at-home PBMt and/or troubleshooting device technical issues;
- Exercising consistently (e.g., brisk walking, weights, Pilates, targeted Parkinson's programs; PWR! Moves or Rock Steady Boxing) a minimum of 3 times per week for a minimum total of 3 hours per week.

Exclusion Criteria

- Previous PBMt for PD
- Cognitive impairment – Montreal Cognitive Assessment (MOCA) score of ≤ 23/30;
- Insufficient understanding of English to sign an informed consent, understand teaching and to perform at-home PBMt;
- Physically unable to perform tasks required for outcome measurement testing
- History of significant musculoskeletal disorders (arthritis or orthopedic injury that would impact outcome measurement testing);
- History of significant neurological disease (stroke, peripheral neuropathy, seizures in the past year)
- Hypersensitivity to light and/or have migraine headaches with aura);
- History of Parkinson's disease in direct family (> 2 family members in the past 2 generations)
- History of significant uncontrolled cardiovascular disease, including heart arrythmia, uncontrolled blood pressure, postural orthostatic tachycardia syndrome (POTS), orthostatic dysautonomia or orthostatic intolerance;
- Significant psychiatric condition (schizophrenia, bipolar disorder, psychotic episodes or suicide ideations in the past 12 months);
- Serious uncontrolled or unstable medical illness (renal, gastrointestinal, terminal cancer);
- Photosensitive Medications (imipramine, hypericum, phenothiazine, lithium, chloroquine, hydrochlorothiazide, tetracycline);
- Corticosteroids or consistent use of anti-inflammatory medication (>2 times/ day);
- Unable to attend PD Wellness & Innovation Centre in Hamilton, Ontario, Canada, for teaching and outcome measurement testing;
- Not actively involved in exercise a minimum of 3 times weekly for a total of three hours weekly;
- Unable to connect for video training sessions at-home PBMt supervision and/or to troubleshooting device technical issues.

Table S2: PBMt Device Parameters

Symbyx PDNeuro LED Helmet

**Light Source:**	20 Red light LEDs of 635nm and 20 near-infrared light LEDs of 810nm
**Treatment Duration:**	24 minutes (12 minutes red; 12 minutes near-infrared)
**Energy delivered / treatment session**:	388 joules
**Pulse Rate:**	40 Hz

Symbyx PDCare Laser

**Laser Class**:	1
**Medical Laser Class**:	2a
**Wavelength**:	superpulsed 904 nm (near-infrared)
**No. of Laser Diodes**:	2
**Pressure Required to Activate Device**:	5 newtons of force
**Average Output Power**:	2 x 30 mW
**Total Output Power**:	60 mW
**Joules delivered per point**	x 2-minute treatment – 7.2 j/cm2
**Energy delivered / treatment session**:	72 joules
**Frequency**:	50 Hz, super pulsed
**Regulatory Approvals**:	SYMBYX PDCare Laser is Australian Register of Therapeutic Goods (ARTG): 335443 and CE marked 0413 as a complementary therapy for the reduction of Parkinson's symptoms.

Table S3: Demographic Characteristics of Study Participants at Baseline

		Active PBMt (N = 32)	Sham PBMt (N = 31)
Age (Mean; SD)		70 (5.98)	69.6 (6.1)
Sex	Male (N)	18	25
	Female (N)	14	6
Years since diagnosis (Mean; SD)		6.3 (3.72)	6.7 (4.84)
Height (Mean; SD)		174.0 (10.03)	173.4 (11.38)
H&Y Stage (N)	1.5	0	1
	2	24	16
	3	6	12
	4	2	2
Weight (Mean; SD)		74.26 (14.451)	81.60 (15.871)
BMI range (N)	< 18.5	2	1
	18.5 - 24.9	13	11
	25 - 29.9	12	8
	≥30.0	1	8
	No data	4	3
Education (N)	High school	8	3
	College graduate	12	15
	Advanced degree	0	0
	No data	0	2
Marital (N)	Single	1	0
	Married/partner	29	26
	Divorced	0	3
	Separated	0	0
	Widowed	2	0
	No data	0	2
Occupation (N)	Service	3	3
	Professional	25	23
	Labor	3	3
	No data	1	2
Work status (N)	Full time	0	2
	Part time	4	4
	Retired	28	21
	Disability/Sick leave	0	1
	No data	0	3

Table S4: Participant Symptom Characteristics at Baseline

Symptom	**Active PBM (N = 32)**	**Sham PBM (N = 31)**
Anxiety	3	8
Balance issues	18	12
Bilateral tremor	9	5
Constipation	14	14
Depression*	2	7
Diarrhea	0	2
Difficulty falling asleep	3	4
Difficulty staying asleep	9	8
Discomfort	0	0
Dyskinesia	4	2
Eye or vision problems	1	2
Fatigue	10	6
Freezing	6	2
Gait issues	7	10
Hallucinations or delusions	1	0
Memory issues	12	11
Muscle cramps	1	2
Pain	0	7
Posture issues	2	2
REM Sleep disorder	1	0
Restless legs	0	2
Rigidity	5	4
Saliva control issues	1	0
Slowness	7	7
Smell reduced or gone	5	3
Speech issues	10	12
Stiffness	2	10
Swallowing difficulty	2	3
Unilateral tremor	12	13
Urinary frequency, urgency, incontinence	6	2
Vivid dreams	0	1

Table S5: Study Withdrawals

Stage	Reason
**Stage 1**<br>4 withdrawals	• one participant consented, baseline assessment completed, did not return, and no explanation was given<br>• one participant had significant covid symptoms,<br>• one participant had a flare-up of previously diagnosed cardiac symptoms with headache and nausea<br>• one participant returned to their respective countries of origin
**Stage 2**<br>8 withdrawals	• one participant returned to their respective countries of origin<br>• one participant death in the family and took an extended holiday,<br>• one participant unrelated surgery, developed an infection, antibiotics and analgesics required<br>• one participant experienced hair loss,<br>• one participant deep brain stimulation insertion after waiting 1.5 years,<br>• two participants refused outcome testing, stating it was too stressful,<br>• one participant reoccurrence of vivid dreams
**Stage 3**<br>8 did not continue	• three participants travel distance/no transportation to the research site<br>• two participants death/illness in the family<br>• one participants general decline related to urinary retention and chronic symptomatic UTIs<br>• two participants started other alternative Parkinson’s treatments
